# Clinical handover communication at maternity shift changes and women's safety in Banjul, the Gambia: a mixed-methods study

**DOI:** 10.1186/s12884-022-05052-9

**Published:** 2022-10-21

**Authors:** Faith Rickard, Fides Lu, Lotta Gustafsson, Christine MacArthur, Carole Cummins, Ivan Coker, Amie Wilson, Kebba Mane, Kebba Manneh, Semira Manaseki-Holland

**Affiliations:** 1 University of Birmingham Medical School, Edgbaston, Birmingham, UK; 2 Institute of Applied Health Research, College of Medical and Dental Sciences, University of Brimingham, Edgbaston, Birmingham, UK; 3 Bundung Maternal and Child Health Hospital, Banjul, The Gambia; 4 Kanifing General Hospital, Banjul, The Gambia

**Keywords:** Handover, maternity, communication, safety, quality improvement

## Abstract

**Background:**

Clinical handover is a vital communication process for patient safety; transferring patient responsibility between healthcare professionals (HCPs). Exploring handover processes in maternity care is fundamental for service quality, addressing continuity of care and maternal mortality.

**Methods:**

This mixed-methods study was conducted in all three maternity hospitals in Banjul, The Gambia. Shift-to-shift maternity handovers were observed and compared against a standard investigating content and environment. Semi-structured interviews and focus group discussions with doctors, midwives and nurses explored handover experience.

**Results:**

One hundred ten nurse/midwife shift-to-shift handovers were observed across all shift times and maternity wards; only 666 of 845 women (79%) were handed over. Doctors had no scheduled handover. Shift-leads alone gave/received handover, delayed [median 35 min, IQR 24–45] 82% of the time; 96% of handovers were not confidential and 29% were disrupted. Standardised guidelines and training were lacking.

A median 6 of 28 topics [IQR 5–9] were communicated per woman. Information varied significantly by time, high-risk classification and location. For women in labour, 10 [IQR 8–14] items were handed-over, 8 [IQR 5–11] for women classed ‘high-risk’, 5 [IQR 4–7] for ante/postnatal women (p < 0.001); > 50% had no care management plan communicated.

Twenty-one interviews and two focus groups were conducted. Facilitators and barriers to effective handover surrounding three health service factors emerged; health systems (e.g. absence of formalised handover training), organisation culture (e.g. absence of multidisciplinary team handover) and individual clinician factors (e.g. practical barriers such as transportation difficulties in getting to work).

**Conclusion:**

Maternity handover was inconsistent, hindered by contextual barriers including lack of team communication and guidelines, delays, with some women omitted entirely. Findings alongside HCPs views demonstrate feasible opportunities for enhancing handover, thereby improving women's safety.

**Supplementary Information:**

The online version contains supplementary material available at 10.1186/s12884-022-05052-9.

## Background

In 2015, The Gambia in West Africa, had an estimated maternal mortality rate (MMR) of 706 maternal deaths per 100,000[1], one of the ten highest worldwide. Since 1990, no significant improvement had been made despite global attention to maternity provisions [1, 2]. The majority of maternal deaths in low-middle income countries (LMICs) are preventable [3, 4]. To combat this, the World Health Organisation (WHO) has increased focus on the quality of maternity care [5], and has highlighted communication failures during patient handovers as one of the top five patient safety problems[6] and a critical component of WHO standards of quality maternity services and women's safety [5]. Hence, optimal communication has been identified as a key strategy to reduce adverse events and improve outcomes [5, 7].

Shift-to-shift clinical handover is a vital communication point for patient information transfer, fundamental to patient safety [6, 8, 9]. It is defined as the transfer of patient responsibility and accountability between collaborating healthcare professionals (HCPs). High-quality handover enables continuity of care between medical providers, facilitating shared awareness of patients' conditions and planned management [10]. Inadequate handovers can lead to delays in diagnosis, treatment errors and life-threatening adverse events [9–15].

Shift-to-shift handover is particularly important within maternity [8, 16, 17]. Complications during pregnancy are frequent and changes in observations and clinical condition occur rapidly affecting both mother and baby [18–20]. In The Gambia and many LMICs, this is exacerbated by the fact that many women in hospital are high-risk pregnancy referrals. Furthermore, the high rate of grand-multiparity (fertility rate of 5.8 births per woman [21]) increases the risk of complications. Many high-income country (HIC) settings still struggle with optimal shift-to-shift maternity handover [22], with studies reporting inconsistent and even inaccurate handovers [23, 24] leading to the implementation of guidelines to standardise handover in several HICs [25, 26]. whilst our systematic review indicates that no research has taken place in LMICs. [27]

Women globally are encouraged to attend health facilities for maternity care from a skilled birth attendant; in The Gambia, 55% of women now attend such facilities to give birth [28]. However, with high workloads, continuity and quality of care can be overlooked, likely implicating maternal and infant mortality; anecdotally handover communication plays an important part in this. These problems in care quality can impact the benefits of health facility birth [16, 17].

Clinical handover procedure is dependent on context, with current practice and culture creating barriers and facilitators to handover unique to specialties, roles and countries [29, 30]. However, important lessons and recommendations for health systems can be learnt by in-depth study of one handover system, developing approaches to measure quality of handover for numerous comparable LMIC's [30].

The aim of this study was to investigate handover practices between HCPs at shift changes on maternity wards in The Gambia; specifically to assess the quality of handover including barriers and facilitators, as defined by environment of handover, staff participation and content of clinical information handed over. Findings will be developed into a conceptual model with specific recommendations which could enhance maternity care in The Gambia and parallel LMICs.

## Methods

### Study design

A mixed-methods cross-sectional study was conducted from January to March 2018 in maternity units in all three government hospitals in the urban Greater Banjul region of The Gambia; Hospital 1 (tertiary), 2 (secondary) and 3 (secondary). The mixed-methods design included both quantitative and qualitative methodology. (See Additional File 1 details of health systems and hospitals).

### Quantitative data collection

Informed written consent was gained to observe staffs (including nurses, midwives, medical officers and doctors) who conduct handover on the maternity ward at shift changes for the timeframe of the study.

An observation tool covering handover content and environment was completed on the wards by three trained UK medical student researchers, each observing one shift at a time (Additional File 2). Environmental factors included: distractions, maintaining confidentiality, location, use of non-technical language, environment supportive of questions, communication method and documentation, time set aside and HCP involvement. As no prior handover standards existed in The Gambia, the observation checklist was adapted from a study in the USA [24] to also include the British Royal College of Obstetricians and Gynaecologists [25] and the American College of Obstetricians and Gynaecologists [26] guidelines. The researchers classified each woman as “in active labour” or not, and either 'high-risk' or not (obstetric emergencies or risk factors for complications as defined by the National Institute for Health and Care Excellence and WHO guidelines [18, 19]). Classification was recorded from information communicated during handover and by asking the staff afterwards. The tool was piloted in The Gambia before use. All handovers were conducted in English. To reduce bias, a similar proportion of morning, afternoon and evening shift changes were observed across the three hospitals, seven days a week, including public holidays. As handovers on different wards occurred simultaneously, researchers moved between wards to attempt to increase observations. Data were recorded onto paper and subsequently digitized using Microsoft Excell.

### Statistical analysis

It was calculated that a conservative sample size of 385 individual women's handovers would detect a 50% prevalence of the handover items communicated with 95% confidence intervals with a 5% margin of error. Information topics (handover contents) recommended for good handover were categorised by Situation (10 items), Background (11 items), Assessment (3 items), Recommendations (4 items) (SBAR) [25, 26, 31, 32]. Non-parametric tests, Mann–Whitney U or Kruskal–Wallis, were used where appropriate for univariate analysis. Multiple linear regression with the number of items handed over as the dependent variable and the independent variables hospital, ward location, time of shift change, day observed, handover lead, whether in active labour, high risk woman, reason for admission, whether questions were asked at handover, whether notes were used, interruptions, handover delay, handover duration and number of women handed over was conducted to identify potential confounding factors from amongst those decided a priori using evidence from the literature. [9, 25, 26] Data were analysed using SPSS version 24.0.

### Qualitative interviews

The qualitative and quantitative components of the study were conducted in parallel. A purposive sampling frame included all cadres of HCPs (nurses, midwives, medical officers and doctors). Methodology combined both focus group discussions (FGDs) to gain diversified perspectives and one-to-one semi-structured interviews (SSIs) to examine personal beliefs and attitudes more comprehensively. The FGDs were challenging as the HCPs were not available at the same time. No staff took part in both FGDs and SSIs. Participants were recruited until thematic saturation was achieved.

One UK medical student trained in qualitative methodology conducted all the interviews. FGDs and SSIs were conducted at the hospital sites using a pre-determined piloted topic guide (Additional File 3). All interviews were conducted in English, spoken by all HCPs in The Gambia. The interviews were recorded, transcribed verbatim and anonymised. Field notes and analytic memos were also made after every interview.

### Data analysis

Inductive thematic analysis based on Braun and Clarke’s six-step approach [33] was undertaken. A second researcher independently double-coded four of the most data-rich transcripts for analyst triangulation [34].

## Results

A total of 110 nurse and midwife shift changes for 666 individual women (see Table 1 and 2) were observed out of a possible 945 shift changes over the 5 weeks in the three hospitals (Additional File 4). The number of women handed over at each shift ranged from 0 to 24, with 179 (21.2%) women on the wards not being handed over. At 5 observed shift changes (4.8%) there was no verbal handover and the staff left before the next staff arrived.

**Table 1:** Characteristics of observed nurse and midwife handover sessions (no planned doctor handover sessions took place in the period of the study)

**Handover characteristics**	
**Total no. nurses** n **Total no. midwives** n **Total no. doctors** n	61 58 42
**No. of shift changes observed total** n (%)	110 (12)
**No. of individual women handed over total** n (%)	666 (79)
**No. of shift changes observed** **Weekdays** n (%) **Weekends/Bank holidays** n (%)	83 (75) 27 (25)
**No. of shift changes observed** n (%)	
**8am**	40 (36)
**2 pm**	39 (35)
**8 pm**	31 (28)
**Duration of handover session in minutes** median (IQR)	4 (3–7)
**No. of handovers delayed > 5 min after shift change** n (%)	90 (82)
**Handover delay in minutes** median (IQR)	35 (24–45)
**No. of shifts where handover not occurred** n (%)	5 (4.8)
**No. of women on each ward** median (IQR)	5 (3–13)
**No. of women handed over on each ward** median (IQR)	4 (2–8)
**No. handover sessions with non-urgent interruptions** n (%)*	32 (29)
**Aids used for verbal handover** n (%)	
**Staff wrote own notes**	54 (8)
**Maternity cards/Ward notes**	566 (85)
**Whiteboard** **Questions asked** **Standard medical terminology used**	52 (8) 168 (25) 646 (97)

^*^non-urgent interruptions; questions from other staff, power cuts, removing relatives, phones ringing, new admissions or finding patient notes

**Table 2:** Frequency of inclusion of each information item on data schedule

**Item on observation tool** *n* = 28	High-risk* Frequency (%) ***n* = 266**	**Active labour** Frequency (%) ***n* = 94**	**Not active labour/high-risk** Frequency (%) ***n* = 347**	**Total** Frequency (%) ***n* = 666**
Situation				
**Lead identified**	266 (100)	94 (100)	347 (100)	666 (100)
**Location**	266 (100)	93 (99)	345 (99)	663 (99)
**Woman's' Name**	177 (66)	68 (72)	236 (68)	445 (67)
**Vital signs**	79 (30)	56 (60)	39 (11)	152 (23)
**Specific concerns about woman**	51 (19)	20 (21)	27 (8)	84 (13)
**Gravidity/Parity**	26 (10)	41 (44)	12 (3)	65 (10)
**Key woman's values**	22 (8)	6 (6)	19 (5)	43 (6)
**Gestation**	14 (5)	14 (15)	3 (1)	23 (3)
**Age**	5 (2)	4 (4)	5 (1)	12 (2)
**Resuscitation status**	9 (3)	5 (5)	1 (0)	10 (2)
Median (IQR)	3 (3–4)	4 (3–5)	3 (2–3)	3 (3–4)
Background				
**Current medications**	135 (51)	20 (21)	112 (32)	253 (38)
**Main complaint**	151 (57)	48 (51)	66 (19)	236 (35)
**Brief history**	117 (44)	47 (50)	91 (26)	229 (34)
**Admission date**	109 (41)	56 (60)	78 (22)	222 (33)
**Diagnosis/Active problems**	111 (42)	38 (40)	61 (18)	182 (27)
**Other chart information**	39 (15)	45 (48)	35 (10)	105 (16)
**Physical examination results**	47 (18)	68 (72)	8 (2)	98 (15)
**Progress from admission**	101 (38)	55 (58)	140 (40)	81 (12)
**Treatment response**	38 (14)	4 (4)	17 (5)	56 (8)
**Laboratory results**	29 (11)	17 (18)	13 (4)	48 (7)
**Allergies**	0 (0)	0 (0)	0 (0)	0 (0)
Median (IQR)	3 (2–4)	4 (3–6)	1 (1–2)	2 (1–4)
Assessment				
**Clinical impression of woman**	137 (52)	54 (57)	177 (51)	344 (52)
**Critical assessment of situation**	54 (20)	26 (28)	23 (7)	85 (13)
**Concerns/problems**	49 (18)	24 (25)	23 (7)	81 (12)
Median (IQR)	1 (0–1)	1 (0–2)	1 (0–1)	1 (1–1)
Recommendations				
**Management plan**	139 (52)	54 (57)	142 (41)	302 (45)
**Time scale**	60 (23)	20 (21)	40 (12)	106 (16)
**Requests/Tests**	50 (19)	22 (23)	34 (10)	92 (14)
**Critical features of management**	44 (16)	14 (15)	12 (3)	59 (9)
Median (IQR)	1 (0–2)	1 (0–2)	0 (0–1)	0 (0–1)
Summary data				
**Median no. items handed over** (IQR)	8 (5–11)	10 (8–14)	5 (4–7)	6 (5–9)
Global comments** n (%)	5 (2)	5 (2)	98 (28)	206 (31)

^*^'high-risk' as defined by National Institute for Health and Care Excellence and WHO guidelines. For breakdown of conditions observed see Additional File 6 and Methods

^**^Global comments included, "normal delivery", "post caesarean section", "stable", "you know her", "no problems" and "continue treatment"

A median of 3 (IQR 2–4) staff changed at each ward shift but only the lead nurse/midwife for the shift received handover as a one-to-one, face-to-face communication from the previous lead. There was no further handover from the shift-lead to the rest of the team except for delegation of specific tasks. Staff read the patient notes or asked the shift-lead if they had questions regarding the women they were caring for.

Doctors were often not based on the maternity wards and did not conduct routine shift-to-shift handovers. Instead, they attended to review women when called, or only communicated specific concerns to the next shift doctor on the phone. Occasionally, ward meetings discussed new admissions. In one hospital, doctors had 24-h on-call rotas which meant they knew about the women admitted.

### Handover environment

Characteristics of the handovers are given in Table 1. Of the 90 (81.8%) delayed handovers, only 4.5% were due to emergencies or situations requiring the shift-lead's attention. Emergencies included complex births and post-partum haemorrhage. Handovers in all hospitals were conducted at the end of each woman's bed except in the labour rooms in Hospital 2 where handover took place in front of a completed whiteboard located in the adjoining staff station (12% of handovers) in view of HCPs, patients and visitors. This displayed women's details including name, gravidity, parity, vital signs and physical examinations. All hospital wards had rooms available where handover could potentially have taken place confidentially, however many were already used for other purposes. No specific written protocols or pro-forma were available to assist with verbal handover. A report book, located at the nursing station on all wards, contained a brief handwritten summary of certain high-risk women on the ward and was updated at the end of each shift.

### Individual handover information content

During verbal handovers, out of a possible 28 topics, a median (IQR) of 6 (5–9) SBAR items were communicated for each woman (Table 2). Handovers commonly discussed more "Situation" and "Background" topics compared to "Assessment" and "Recommendation'' topics, where in around half of handovers, no information was exchanged (see Additional File 5). No SBAR items were consistently communicated for all women (except "lead identified" and "patient location" which were implied with the bedside handover style).

Women in active labour versus not in active labour had a median of 4 more information items discussed. Those deemed high-risk also had significantly more items communicated (Tables 2 and 3). The fewest items were discussed in ante and postnatal wards, where 196 (29%) women were handed over with only a global comment.

**Table 3:** Handover characteristics and associated predicted number of items handed over in multiple regression analysis

Handover characteristics	Frequency (%) *median (IQR)	Median (IQR) Items	*p* value	Regression coefficient (95% CI): change in n of handover items discussed	*p* value
Hospital					
Hospital 1	190 (28)	5 (4–8)		-0.82 (-1.54 to -0.09)	0.028
Hospital 2	172 (26)	8 (5–11)	< 0.001***	1	
Hospital 3	304 (46)	6 (5–9)		0.11 (-0.48 to 0.70)	0.720
Ward Location					
Labour ward	104 (16)	11 (8.-14)		1	
High Dependency Unit	99 (15)	8 (6–11)	< 0.001***	-0.83 (-1.91 to 0.25)	0.130
Ante/postnatal ward	463 (69)	5 (4–7)		-1.77 (-2.85 to -0.68)	0.001
Time of shift change					
8am	311 (47)	6 (5–8)		-0.84 (-1.41 to -0.27)	0.004
2 pm	196 (29)	7 (5–12)	< 0.001***	1	
8 pm	159 (24)	7 (5–10)		0.15 (-0.50 to 0.80)	0.964
Day observed					
Weekday	498 (75)	6 (5–9)	0.130**	1	
Weekend/Bank holiday	168 (25)	7 (4–9)		-0.27 (-0.76 to 0.22)	0.277
Handover lead					
Midwife	253 (38)	6 (5–9)		1	
Nurse	412 (62)	6 (5–9)	0.230**	0.43 (-0.10 to 0.96)	0.115
Active labour					
No	572 (86)	6 (5–9)		1	
Yes	94 (14)	10 (8–14)	< 0.001**	1.06 (0.12 to 1.99)	0.027
High-risk woman					
No	400 (60)	6 (5–9)		1	
Yes	266 (40)	8 (5–11)	< 0.001**	1.30 (0.79 to 1.82)	< 0.001
Reason for admission					
Labour	615 (92)	6 (5–9)		1	
Observation	51 (8)	7 (5–12)	0.411**	-1.01 (-1.83 to -0.20)	0.015
Questions asked at handover					
No	168 (25)	6 (4–9)		1	
Yes	498 (75)	7 (5–10)	< 0.001**	1.11 (0.58 to 1.64)	< 0.001
Notes used at handover					
No	612 (92)	6 (5–9)		1	
Yes	54 (8)	6 (5–9)	0.118**	0.12 (-0.65 to 0.89)	0.761
Interruptions					
No	609 (91)	7 (5–9)		1	
Yes	57 (9)	5 (8–4)	< 0.001**	0.23 (-0.56 to 1.02)	0.573
Handover delay					
Time after official shift change (mins)	35 (24–45)*	-	-	0.02 (0.01 to 0.03)	0.002
Handover duration					
Length (mins)	4 (3–7)*	-	-	1.99 (1.46 to 2.53)	< 0.001
Number of women handed-over					
Number of women	4 (2–8)*	-	-	0.08 (0.03 to 0.14)	0.003

^**^Mann–Whitney U test

^***^Kruskal–Wallis test

### Comparison of handover content

On multivariate linear regression analysis (Table 3), the independent variables that significantly influenced the number of items handed over included the following; with fewer items discussed at the ante/postnatal ward, at the early shift change, tertiary hospital and more discussed where were women were in active labour or high-risk, admission for observation, when questions were asked and when handovers lasted longer. The regression predicted 46% of the variation in information communicated (R^2^ = 0.46, p < 0.001).

### Qualitative results

SSIs were conducted with eight doctors, eight midwives and five nurses. Two FGDs were conducted with five midwives and four nurses in Hospital 1. Additional File 7 summarises the demographic details of all 30 participants.

Three major themes identifying barriers and facilitators to optimal handover emerged from thematic analysis these included healthcare systems factors, organisational cultural factors and individual HCP factors. These themes along with their supporting quotations are displayed in Table 4. A summary of the salient points is given below.

**Table 4:** Themes with supporting quotations showing barriers and facilitators to effective shift-to-shift handover

Theme	Concept	Supporting Quotations
Health systems / hospital systems factors	Facilitator • Some tools and procedures for effective handover Barrier • Absence of confidentiality of notes • Absence of protocols and standards • Absence of formalised handover training • Poor salary • Extreme staff shortages on the ward • Absence of electronic based record system which could help legibility and rapid information exchange	“we have a board where we write the precaution to the incoming staff that this patient is high-risk or critical or needs attention so as a reminder, the board is there to remind the staff that look you have to do these things.” (M6) [F] “[For] high-risk patients, we do make a special handing over… I use a red pen to outline that information [in patient’s notes] … when they [the HCPs] see that red column, they call their attention more immediately.” (D3) [F] “the manual book that we write the data in… someone can just come in from another ward and open the book [B]… but if it is a database, you can have a code or like a password so it is only us that can get access to the patient data.” (M2) [F] “we don’t have a written guideline…having a protocol also gives you a more clear view of what you actually need for the patient need to do for that patient and what emphasis to put on patients’ care.” (D5) [B] “if we have that [formal training of handover] from medical school or nursing school that would be fantastic. Because then at least we would all be speaking at the same level… so that once we finish school, it’s going to be easy for everybody than coming into the system and then they have to train you all over.” (D6) [B]“the salary is very small… so most of the staffs here, they work in two places, they work in the government here on morning shift. When they close, they go to the private for afternoon… you have to work double shift, which is affecting the handing over.” (M13) [B] “Main problem of the handing over here… is short of staff... the only thing that can improve our handing over is when we have staffs on the ground. So when this one is doing the handing over, others are listening so if this one forgot some information, then the others will remember this one said this and this one said this.” (M1) [B] “We need enough staff, that is now a problem… I have more than like… 10 or 20 patients… so sometimes when we are taking over in the morning, you have a full ward and it is only one midwife-senior midwife that is taking over. You have a lot of information to… to remember, to put in your head and sometimes you tend to miss one or two information” (M2) [B] “I was working in a clinic where it’s all electronic… So rather me struggling to read the handwriting [B], if I have that it’s easy… there will not be any delay in the information… you don’t have to worry about the folders getting torn or you losing the papers… a lot of time will be saved.” (D6) [F]
Organisational cultural factors	Facilitator • Monitoring of handover practice Barrier • Absence of multidisciplinary team handover • Incomplete documentation for handover • HCPs not performing a detailed handover to the next shift • Healthcare staff tardiness	“There are days that the handing over may not be done appropriately but we, from time to time, we pop in and see what they have been doing so as the one who supervises them, I have to be on their toes from time to time to see whether the handing over is being done or not.” (D4) [F] “Nurse will hand over to the nurse, mostly doctors will hand over to the doctors. It’s all separate.” (N5) [B]“…and not all the patients are even written in the book….” (M13) [B] “there is no proper documenting… you may have done something for a patient, you did not record it… the one who hand over may come and… give the same medications to the same person.” (N7) [B]“Sometimes even the handing over is not done. You only write report and leave the book there. When they come, they read from the report book… … we only report special cases and you leave the rest because we are rushing to leave… [B] “If I am in a shift and a nurse didn’t come until after one hour, the nurse is late, I leave the patients.” (M13) [B] “when you have to hand over and then the doctor that is supposed to come is a bit late. You have to sit and wait until she comes or he comes… that is affecting the handing over because you have to go home, you have another activity to do.” (D2) [B] “most of the time when they come in the morning, either the night staff are rushing to go home or the morning staff are late, so they don’t usually hand over properly.” (D6) [B]
Individual healthcare professional factors	Facilitator • HCP knowledge and experience of importance of handover in patient safety Barrier • Practical barriers e.g. difficulty with transport getting to work • Absence of childcare facilities in close proximity to the hospitals • Illegible handwritten notes • Many HCPs are computer illiterate • HCPs working in multiple places and working double shifts	“I’ve experienced, if you don’t do proper handing over, it leads to some problems. Lapses are made about a patient.” (M13) [B] “there is no proper documenting… you may have done something for a patient, you did not record it… the one who hand over may come and… give the same medications to the same person.” (N7) [B]“the only way to solve it [of HCP tardiness] is the hospital to provide transport for the staff… everyone struggle on their own… you will stand one hour, two hours just to have a car to go home, so it’s a problem and the salary is not that much for you to hire a taxi every day.” (M12) [B] “here, we don’t have a centre where you put your kids, a day care… So I think also it could be part of solving the problem [of HCP tardiness], if we had a centre for mothers here where you can put your child.” (M13) [B] “… also the other thing is that their handwriting is bad… you can’t read… Handwriting is important because you’re writing for someone to read so if you’re writing it and someone else can’t read it so it’s useless, it’s like don’t write.” (D7) [B] “Not everyone has basic skills in this computer…it [an electronic system] would be advantageous for us, the ones that have skills on it, but the ones that don’t have skills on it, they will not even look at it, they will not even bother themselves.” (N5) [B] “And sometimes you either work on afternoon here then go to private at night, so like for example, those on morning shift here, they are always in a haste… to go early to that other hospital they are working… Because you that feel here, the salary is not much for you, you have to work double shift, which is affecting the handing over.” (M13) [B]

Abbreviations: The code letter after each quotation refers to the cadre of HCP; doctor (D), midwife (M) and nurse (N). [F] illustrates a facilitator; [B] shows a barrier

#### Health systems factors

Specific handover practices to facilitate effective handover for high-risk patients were described, such as, the use of a whiteboard to inform the next shift about patients who require extra care. A commonly reported barrier to clinical handover was the extreme staff shortages on the maternity wards. Consequently, handovers were often carried out in pressurised work environments with important clinical information being omitted. Many participants spoke of how the absence of formal protocols and training resulted in a wide variation in the quality and content of handovers, thus were highly supportive of implementing standardised protocols.

#### Organisational cultural factors

Many barriers to handover were identified. The majority of HCPs expressed a need for a whole team handover. The high patient-to-staff ratio and a large amount of information being passed on from one shift to the next meant that the lead-nurse was often overloaded with information that was difficult to retain. Furthermore, many participants spoke of the inadequacies of the documentation culture. They reported that “incomplete notes” coupled with “illegible handwriting” could lead to information lapses regarding patient care. A few respondents discussed how these communication failures had resulted in the omission or duplication of treatments, potentially causing patient harm. A small number of respondents expressed concern about how seemingly stable patients could often be missed during handovers as HCPs focused their attention on patients categorised as more critically ill. The majority reported a lack of punctuality of staff for their shift handovers. Participants discussed how this can jeopardise patient safety, for example, “wards can be left unattended when one set of staff have to leave before the next shift arrive”.

#### Individual HCP factors

Important facilitators were that most of the staff were aware of the importance of handover. Transportation difficulties, frequently resulting in staff tardiness, were repeatedly mentioned as a barrier to effective handover. Participants also mentioned social factors, including the shortage of childcare provisions in close proximity to the hospitals, as an additional barrier.

### Mixed method triangulation

**Fig. 1:**
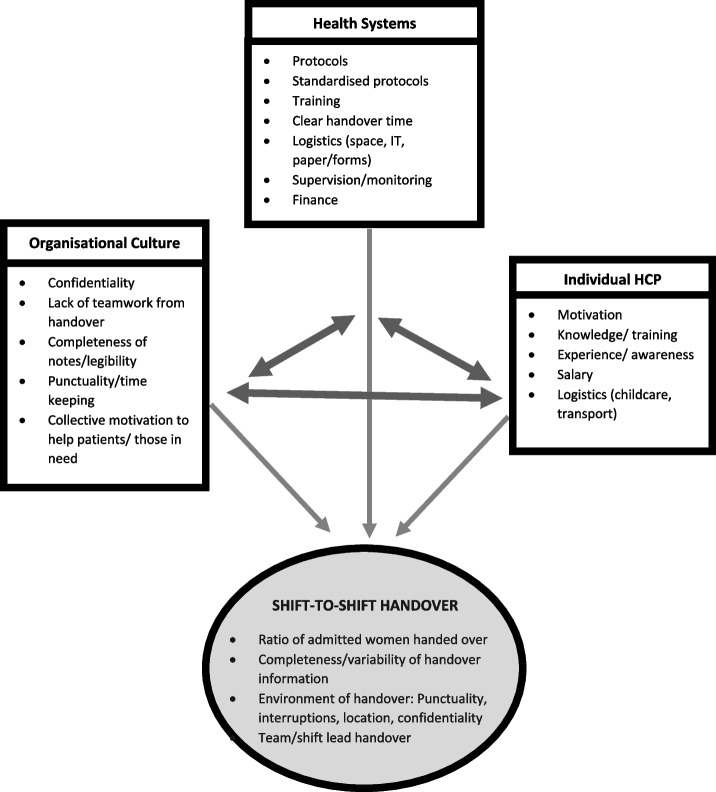
Conceptual diagram for Influential Factors—Model showing triangulation of interaction between health systems, organisational culture and individual HCP factors, and their contributing influence on maternity handover

The quantitative and qualitative results were triangulated in order to guide intervention development [35]. The health systems, organisational cultural and individual HCP factors that interact and influence effective handover as barriers and facilitators, in our setting were applied to develop a conceptual diagram (Fig. 1). For example: need for standardised protocols is recognised by motivated individuals, health systems are required to drive development, with organisational support essential to address standardisation, use of tools and promoting inclusion of all women in shift-to-shift handover.

## Discussion

Information communication at handover presents a major patient safety challenge globally [6]. Based on set criteria, we assessed the rate of women handed over, the information content and environment of handover in all labour, high-dependency, antenatal and postnatal wards in all public maternity hospital units in The Gambia's capital city to establish the facilitators and barriers to optimal handover. Qualitative and quantitative data supported and expanded on findings; despite no formal handover protocol, training or monitoring the concept of handover, including patient safety aspects and need for confidentiality, was widely understood and valued by HCPs. However, numerous aspects of shift-to-shift handover need attention in order to reduce errors experienced by HCPs and improve continuity of care. These involved the lack of full staff participation, missed handover sessions or missed women, handover environment, variable and scanty clinical content communicated.

We have translated the research findings into a framework of systems culture, organisational culture and individual HCP factors. These themes identified aligned with those in the Australian Commission on Safety and Quality in Health Care [36] and Humphries et al. in India [37]; indicating that the framework embraces factors applicable to maternity units in other LMICs alongside other specialities. The following discussion utilises this foundation to develop interventions and recommendations supporting wider maternity service assessment and care improvement.

### Standardisation protocols and training

The important obstacles to most aspects of optimal handover were health systems factors, primarily lack of standards, protocols and training which were strongly supported by our HCPs. Studies in HICs alongside WHO standards for HICs and LICs, currently advocate guidelines and pro-forma for maternity handovers; applying tools such as SBAR for efficient communication and reducing adverse events. A simple checklist would further support information retention in staff who felt overloaded with information [5, 25, 26, 38]. A continuum of culturally-sensitive training, formal and informal, running alongside behaviour change methods (such as non-monitory incentives, handover champions, team supervision and monitoring) focussed on increasing the impact of handover, is needed to embed handover processes with the aim of improving patient outcomes [39]. These highlight low-cost and sustainable solutions [5, 30, 40, 41]. Staff awareness and experience, verbalising the potential dangers of inadequate or missed handovers, is a vital individual factor facilitating these recommendations and to improve a patient safety culture prioritising handover communication.

### Clinician team participation

Other health systems barriers to adequate handover existed in that doctors have no set handover routine, obtaining information through asking on-ward staff, patients' notes or calling doctors on the last shift. Furthermore, for nurses/midwives, only the shift-leads gave and received handover which was not formally passed on to the rest of the team. This limits proactive monitoring/treatment of women, amplifying the risk of adverse events with potentially critical gaps in clinical information transferred to the majority of HCPs [7]. As suggested by our participants, promoting engagement of all junior and senior staff in handover could improve teamwork, quantity of information communicated[8] and provide valuable learning opportunities for junior staff to confidently support optimal handover culture [39, 42]. However, practically implementing a multidisciplinary team handover in these busy maternity hospitals would require a change in organisational culture with support and staffing [8].

### Location-specific handover with inclusion of all women

Critically over 20% of women were missed from handover, and most others had little clinical information communicated. The WHO recommends in the interest of patient safety [43], quality improvement and optimal pregnancy outcomes, a standardised handover with clear, accurate information transfer should be performed and promoted for all pregnant women [40]. Co-ordinated care for every woman is arguably a priority safety concern in sub-Saharan Africa (SSA) in order to reduce the persistently high maternity death rates [1, 5, 44]. In The Gambia and SSA, many women admitted in maternity wards are high-risk (40% in our study with many referred from local health centres). In active labour, but also ante-natal and post-partum stages, these women by definition need close monitoring for unexpected changes in condition [5, 8]; with over half of maternal and newborn deaths occurring in the first few days post-partum [20, 44]. Health systems based intervention, addressing specific communication requirements in different stages, utilising targeted tools such as the WHO Safe Childbirth Checklist, could reduce handover variability and missed women, guiding continuity of care.

### Delays and interruptions

Lack of protocols led to a number of downstream barriers to comprehensive handovers. With no set times for shift-to-shift handover, timing varied largely leading to absence of either coming or leaving staff or shortened handovers, with some handovers skipped completely. Exacerbated by staff shortages, this was a major barrier blurring the transition of patient responsibility [41]. At the HCP level, prompt handover was not prioritised and significant individual barriers such as finding childcare and transport to and from work were identified. Provision of transport services for staff alongside availability of childcare centres around the hospitals were suggested and could be paid for by the HCPs (as they currently bear the cost) are potential facilitators to punctuality. These organisational interventions, could improve staff motivation and encourage HCPs’ full and equal engagement in work [45, 46].

Compared to other times, 8am handover had fewer information items communicated. Fatigue from 12-h shifts alongside handover delays may have played an important role in this [46]. Interruptions during handover were frequent and acceptable from organisational culture perspective. This is a recognised cause of shortened, inaccurate or incomplete handovers, exacerbated by lack of protected time and staff shortage [47, 48]. Addressing these systems based standards, monitoring a protected handover time and prioritising attendance over other tasks (excluding emergencies) could avoid delays, improving information transfer [29].

### Verbal versus written handover practices

Handovers were commonly verbal only. Alongside a lack of standardised handover documentation and variable legibility of notes instead left staff reportedly relying upon memory or judgement for patient care. This was viewed as an organisational culture issue, where the importance of complete and legible notes was not a priority, even though many staff reported the likely result of errors in care [14, 15, 25, 49]. Practices including writing notes on scraps of paper and the report book (located at all nursing stations containing a brief handwritten summary of some high-risk women, updated each shift) was not unfamiliar and could be built upon to facilitate handover.

Whiteboards were one method employed successfully in one ward to provide handover structure and clarity. For the larger wards (30 beds), creative use of boards needs to be considered [50, 51]. Adoption of electronic databases has been shown to increase the efficiency of information exchange and was supported by our HCPs [52]. However, unreliable electricity supply, funds for establishing such a system, alongside varying HCP computer literacy, are barriers to this solution currently in The Gambia’s health system.

### Promoting service utilisation in LMICs

Reflecting on handover beyond simply provision of care, handover is intricately linked with patient satisfaction and patient-centred care [5, 47]. Holistic concepts (patient values and concerns) were scarcely discussed and all but 4% of handovers were not confidential [48]. This was an organisational culture issue with little prioritisation given to patient-centred care, likely given the clinical pressure of work and lack of human resources. For example, all hospital wards had private/staff rooms available where handover could potentially have taken place confidentially, however these were often used for other purposes. In a country where only 55% of women attend health facilities for childbirth, improving women's experiences has the potential to encourage women to seek healthcare, improving national maternity outcomes [4, 28].

Challenges to adequate handover as demonstrated in this LMIC are ongoing globally across maternity services [8, 22–24, 47]. Handover interventions proposed utilise a low-cost, culturally-sensitive, multi-faceted approach (Table 5). This involves health system managers alongside staff participation with activities that prioritise patient-safety and patient-centred culture [5, 7, 8, 41, 43]. Importantly, our qualitative data demonstrated HCPs’ willingness and drive to provide high-quality care and improve skills. However, clear evidence and consensus on specific information and environment required at maternity handovers requires further development [8, 23, 24, 38, 47, 53]. Our framework (Fig. 1) could begin to provide a conceptual guide for this process.

**Table 5:** Shift-to-shift handover recommendations

Factors	Proposed improvements
**Healthcare systems**	Developing targeted national handover guidelines + re-audit to refine situational appropriateness
	Implement handover training within educational curricula + ongoing professional development
	Consider solutions to staff shortages and salary driving HCPs to work at multiple jobs
	Further assess the role of handover in quality of patient care with further research into the links proposed
**Individual clinicians**	Facilitate work attendance and punctuality by considering transport and childcare options
	Encourage and support HCPs existing awareness and recognition of the importance of handover
	Address note taking and written handover including the consideration of handwriting legibility
**Organisational**	Encourage full team participation in handover for HCPs training, teamwork and accurate handover
	Support handover of all women to minimum criteria as supported by guideline development
	Consider the impact of incomplete documentation and the availability of resources for written handover
	Address the organisational tardiness culture alongside improvements linked to developing handover protocol and approaching the individual factors as above

### Strengths and limitations

This study reports the first research on handover practices in maternity care in Africa. Given the high MMR in SSA, this is a significant contribution to maternity care improvement. Although handover is location, context and culture specific, important lessons and recommendations for comparable LMIC's can be learnt by in-depth study of one handover system such as this. Further research should investigate rural regions of The Gambia and other LMICs to increase generalisability and transferability.

This study minimised selection bias by providing observations across all three-government maternity hospitals in the capital city; researchers observed all maternity wards across a random selection of days and times, including weekends and public holidays. By further triangulating quantitative and qualitative data, the richness of data was maximised to create a versatile framework.

Although the majority of the SSIs were conducted in private, some were carried out on the ward (HCPs unable to leave the ward unattended) which could have compromised participants speaking freely. Quantitative data analysis was limited by the observation tool placing equal weighting on each SBAR information item. Some items may have greater impact on patient care, particularly across different wards. Additionally, researcher presence could have influenced handover, with some HCPs conducting a more detailed handover and others omitting information. However, we consider the large sample size would have diluted these effects. Furthermore, classification of high-risk relied on information given at handover and clarifying with HCPs afterwards. This may have overestimated the quality of information discussed for this group since some high-risk women may not have been identified. The checklist used was adopted from a study in USA as we could find nothing similar from a LMIC setting. The qualitative findings however supported use of the checklist as HCPs demonstrated and volunteered the importance of similar principles, facilitating the development of specific and targeted recommendations.

## Conclusion

Clinical shift-to-shift handover in maternity care is a critical communication moment with major implications for maternity care safety globally. This study has identified culturally-sensitive recommendations to address the complex barriers to effective communication in one sub-Saharan African capital city, recommending that it should become routine to discuss all women at handover (even if briefly) and reducing information omission, particularly for antenatal and postnatal women. Alongside this, investigating innovative methods to improve punctuality and to implement a protected handover time/location is an important next step. Identification of standardised priority topics to be communicated in different locations will aid the development of specific guidelines and reduce communication lapses. We advocate a multi-level approach prioritising systems approaches such as hospital policy alongside formalising handover guidelines, monitoring and training in The Gambia. Importantly, much of the factors affecting handover visualised in our framework are also relevant to other acute specialities and wards in LMICs and add to the generalisability of our findings in hospital settings.

## Supplementary Information

**Additional file 1.** Hospital settings and background information.

**Additional file 2.** Quantitative Data observation tool.

**Additional file 3.** Interview Topic Guide.

Supplementary file4. Graph showing spread of observations across days of week, hospitals and shift changes.

**Additional file 5**. Table of total SBAR items discussed at handover.

**Additional file 6.** Table of high-risk and obstetric emergency conditions observed.

**Additional file 7.** Table of characteristics of participants in SSIs and FDGs.

## Data Availability

All dataset used and analysed in this paper are available from the corresponding author on reasonable request.
