# Supplementary material for: Clinical handover communication at maternity shift changes and women's safety in Banjul, the Gambia: a mixed-methods study
Source: BMC Pregnancy Childbirth. 2022 Oct 21;22:784. doi: 10.1186/s12884-022-05052-9 (PMC9587588; doi:10.1186/s12884-022-05052-9)
Supplement: Supplementary file 1 — Additional file 1. Hospital settings and background information. [file 12884_2022_5052_MOESM1_ESM.pdf]

Additional File 1: Hospital settings and background information

There are a variety of health facilities including health centres, government and private hospitals where women in The Gambia can attend to give birth with the support of a trained birth assistant. In Banjul however, there are only 3 government hospitals and alongside smaller health centres for labour with no surgical facilities. Therefore, health centres may provide antenatal care and will tell higher-risk mothers to go to a hospital to give birth. Primary healthcare centres can refer women to any of the three hospitals, normally the closest maternity unit geographically. Women experiencing complications in hospitals in rural provinces further inland ('upcountry') are sometimes referred to Hospitals 1 or 3 in our study.

Maternity wards in the hospital included 20-40 combined antenatal and postnatal beds, 6-8 labour room beds in each hospital and 8-10 high-dependency beds in hospital 1 & 2.

Handovers took place separately in ante/postnatal wards, labour rooms and HDUs in each hospital. As multiple handovers occurred simultaneously, the researchers moved between the wards to attempt to observe as many handovers as possible.

Shift changes occurred at 8am, 2pm and 8pm for all hospitals for nurses and midwives with no regular handovers for doctors and medical officers.

Hospital 1	Hospital 2	Hospital 3
The only tertiary hospital in The Gambia, providing specialist and emergency obstetric care	A maternity specific facility in a local community setting offering emergency obstetric care	The second largest hospital in The Gambia in the centre of the urban coastal area offering emergency obstetric care
Highest referral rate - receives complex cases	Low referral rate - refers complex cases to hospital 1 & 3	High referral rate - Refers complex cases to hospital 1
4 maternity wards:<br>Antenatal, postnatal, labour (open ward with curtains separating beds)and HDU	3 maternity wards:<br>Combined ante/postnatal, labour (private rooms) and HDU	2 maternity wards:<br>Combined ante/postnatal and labour (open ward with curtains separating beds)
56 beds including (8 on HDU)	33 beds (10 on HDU)	28 beds (no HDU)
Maternity staff:<br>33 doctors (the largest number of doctors in any hospital in the Gambia)<br>27 midwives<br>29 nurses	Maternity staff:<br>4 doctors<br>12 midwives<br>20 nurses	Maternity staff:<br>5 doctors<br>19 midwives<br>12 nurses<br>12 nursing assistants
Private spaces where handover could potentially occur include staff rooms or conference room (not directly located on the wards)	Private spaces where handover could potentially occur include small sisters office and staff room	Private spaces where handover could potentially occur include small sisters office and curtained bay at end of labour ward
