# Supplementary material for: Clinical handover communication at maternity shift changes and women's safety in Banjul, the Gambia: a mixed-methods study
Source: BMC Pregnancy Childbirth. 2022 Oct 21;22:784. doi: 10.1186/s12884-022-05052-9 (PMC9587588; doi:10.1186/s12884-022-05052-9)
Supplement: Supplementary file 2 — Additional file 2. Quantitative Data observation tool. [file 12884_2022_5052_MOESM2_ESM.pdf]

Additional File 2: Data Observation Tool

Observation of Maternity Handover **CONFIDENTIAL**

Date: .... **Day of week:** Sun Mon Tues Wed Thu Fri Sat

Time of handover: .... **Duration (minutes):** ....

Location of handover: .... **Hospital:** ....

**No. of patients handed over**		**No. of patients on ward**	
**No. of patients high-risk on ward**		**No. of patients with critical conditions on ward**	
**No. of staff present**		**No. of staff on ward**	

Job titles of Health care professionals present:

	Please Circle:	Additional comments:
**Handover delayed**	YES/NO/NA	
**Emergency during handover**	YES/NO/NA	
**Lead nominated (job role)**	YES/NO/NA	
**Information repeated to ensure accuracy**	YES/NO/NA	
**Access to lab results, notes, intranet**	YES/NO/NA	

Communication method: Verbal Face-to-face / Verbal Phone / Written only / Mixed Verbal-Written

If handover notes provided: Handwritten / Electronic / Other

Any other comments:

Answer with Y/N unless otherwise indicated

Patient number		1	2	3	4	5
Patient is in active labour						
Patient is high risk*						
Reason for admission (Labour/Observation)**						
**Situation**	Woman's name					
	Woman's age					
	Gravidity/Parity					
	Gestation					
	Leading consultant/nurse indentified					
	Patient Location					
	Vital Signs					
	Resuscitation Status					
	Specific concerns					
	Key patient values/needs					
	**Total /10**					
**Background**	Date of admission					
	Present chief complaint					
	Diagnosis/ active problems					
	Brief history					
	Current Medications					
	Allergies					
	Laboratory Results					
	Physical examination results					
	Progress during admission					
	Response to treatment					
	Other information from charts					
	**Total /11**					
**Assessment**	Clinical impression					
	Critical assessment of situation					
	Expression of concerns/problems					
	**Total /3**					
**Recommen-dations**	Management plan					
	Suggestions on requests/ tests					
	Suggestions on time frame					
	Critical management features recognised					
	**Total /4**					
Global Comment ***						
Distractions/Interruptions						
Confidentiality maintained						
Questions asked						
Written notes provided						
Standardised medical terminology						

*Please complete reason for high-risk ** Please answer as follows L=Labour O=Observation

***Specific comment require
