# Supplementary material for: Clinical handover communication at maternity shift changes and women's safety in Banjul, the Gambia: a mixed-methods study
Source: BMC Pregnancy Childbirth. 2022 Oct 21;22:784. doi: 10.1186/s12884-022-05052-9 (PMC9587588; doi:10.1186/s12884-022-05052-9)

Additional File 4: Graph showing spread of observations across days of week, hospitals and shift changes

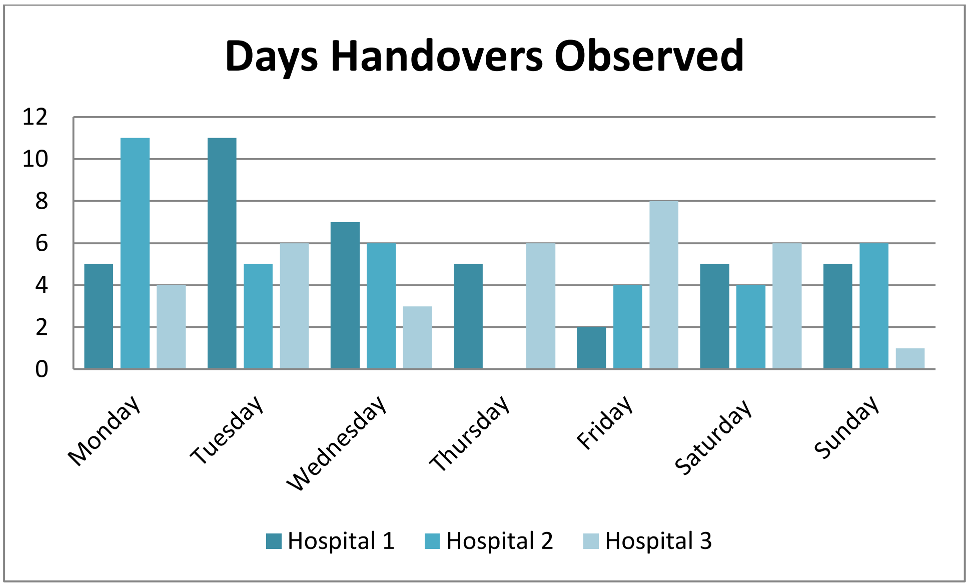

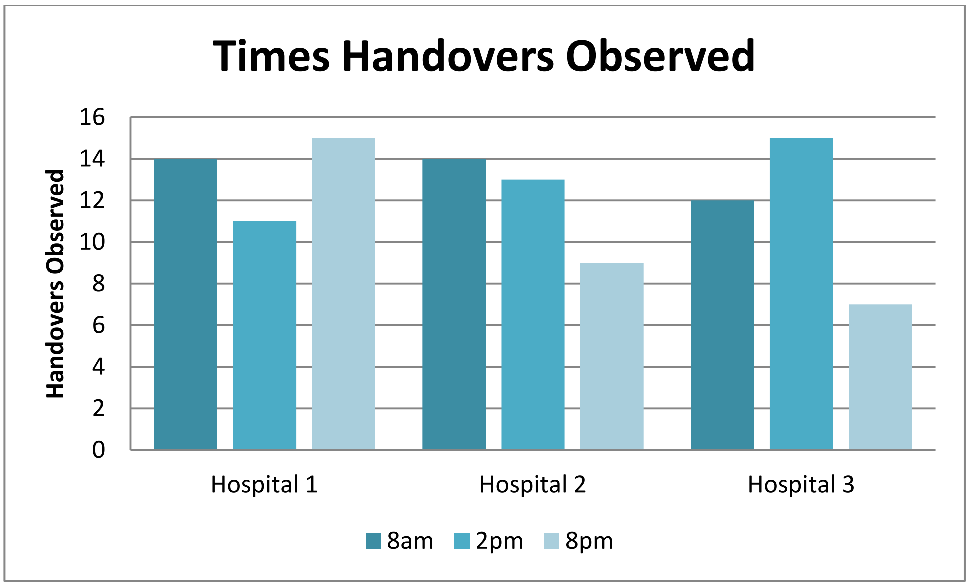

Supplement: Supplementary file 4 — Supplementary file4. Graph showing spread of observations across days of week, hospitals and shift changes. [file 12884_2022_5052_MOESM4_ESM.pdf]
