# Supplementary material for: Clinical handover communication at maternity shift changes and women's safety in Banjul, the Gambia: a mixed-methods study
Source: BMC Pregnancy Childbirth. 2022 Oct 21;22:784. doi: 10.1186/s12884-022-05052-9 (PMC9587588; doi:10.1186/s12884-022-05052-9)
Supplement: Supplementary file 5 — Additional file 5. Table of total SBAR items discussed at handover. [file 12884_2022_5052_MOESM5_ESM.pdf]

<u>Additional File 5: Table of total SBAR items discussed at handover</u>

Table of median number of SBAR items included in all handovers

	**Total (28 items)**	**Situation (10 items)**	**Background (11 items)**	**Assessment (3 items)**	**Recommendations (4 items)**
**Items included** median (IQR)	6 (5-9)	3 (3-4)	2 (1-4)	1 (1-1)	0 (0-1)
**Minimum**	2	1	0	0	0
**Maximum**	22	7	9	3	4
**No items included** frequency (%)	0 (0)	0 (0)	77 (11.6)	298 (44.7)	351 (52.7)
**All items included** frequency (%)	0 (0)	0 (0)	0 (0)	46 (6.9)	21 (3.2)
