# Supplementary material for: Clinical handover communication at maternity shift changes and women's safety in Banjul, the Gambia: a mixed-methods study
Source: BMC Pregnancy Childbirth. 2022 Oct 21;22:784. doi: 10.1186/s12884-022-05052-9 (PMC9587588; doi:10.1186/s12884-022-05052-9)
Supplement: Supplementary file 6 — Additional file 6. Table of high-risk and obstetric emergency conditions observed. [file 12884_2022_5052_MOESM6_ESM.pdf]

Additional File 6: Table of high-risk and obstetric emergency conditions observed

Table displaying high-risk conditions and obstetric emergencies as defined by NICE Intrapartum Care for Healthy Women and Babies Guideline and WHO that were identified during observation of handovers

High-Risk Conditions	Frequency of high-risk conditions (%) n=280*
Pre-eclampsia	63 (22.5)
Previous Caesarean Section	25 (8.9)
Eclampsia	24 (8.6)
Prolonged second stage of labour	19 (6.8)
Pregnancy induced hypertension	17 (6.1)
Multiple birth	17 (6.1)
Anaemia	16 (5.7)
Intrauterine foetal distress	14 (5)
Postpartum haemorrhage	13 (4.6)
Abruption	12 (4.3)
Induction of labour	7 (2.5)
Antepartum Haemorrhage	7 (2.5)
Septic caesarean wound	7 (2.5)
Parity >4	6 (2.1)
Placenta praevia	6 (2.1)
Malpresentation Breech/Transverse lie	6 (2.1)
Cervical cancer	4 (1.4)
Preterm labour	3 (1.1)
Cardiac disease/hypertensive disorders	3 (1.1)
Previous unexplained stillbirth	2 (0.7)
Prolapse of cord	2 (0.7)
Poly/oligohydraminos	1 (0.4)
Previous ectopic	1 (0.4)
Malaria	1 (0.4)
Sickle cell	1 (0.4)
Diabetes	1 (0.4)
Attempted suicide	1 (0.4)
Prolonged third stage of labour	1 (0.4)

*14 women had multiple high-risk conditions and are included twice - 266 individual women had high-risk conditions
