# Supplementary material for: Clinical handover communication at maternity shift changes and women's safety in Banjul, the Gambia: a mixed-methods study
Source: BMC Pregnancy Childbirth. 2022 Oct 21;22:784. doi: 10.1186/s12884-022-05052-9 (PMC9587588; doi:10.1186/s12884-022-05052-9)
Supplement: Supplementary file 7 — Additional file 7. Table of characteristics of participants in SSIs and FDGs. [file 12884_2022_5052_MOESM7_ESM.pdf]

Additional File 7: Table of characteristics of participants in SSIs and FDGs

Demographics of participants in SSIs and FDGs

**Characteristic**	**Categories**	**Number of Participants n= 30**
**Age**	25 and under	3
	26-35	17
	36-45	7
	46-55	2
	56 and over	1
**Gender**	Male	10
	Female	20
**Profession**	Doctor	8
	Midwife	13
	Nurse	9
**Years of clinical experience**	0-5	15
	6-10	5
	11-20	8
	21 and over	2
**Religion**	Muslim	25
	Christian	5
**Ethnic group/ tribe**	Mandinka	10
	Fula	8
	Wolof	3
	Manjago	3
	Other	6
